# Parasympathetic Dominance Decreases the Choroidal Blood Flow Velocity Measured Using Laser Speckle Flowgraphy

**DOI:** 10.7759/cureus.46996

**Published:** 2023-10-13

**Authors:** Fuka Kuwahara, Yuki Hashimoto, Natsumi Toh, Sakurako Imabayashi, Ami Sakamoto, Kanon Shiraishi, Rena Igawa, Takeshi Yoshitomi

**Affiliations:** 1 Department of Orthoptics, Fukuoka International University of Health and Welfare, Fukuoka, JPN

**Keywords:** parasympathetic activity, warm water immersion, mean blur rate, laser speckle flowgraphy, choroidal circulation hemodynamics

## Abstract

Background: This study aimed to examine the changes to choroidal blood flow velocity using laser speckle flowgraphy (LSFG) in healthy eyes after warm water immersion at 40°C.

Methods: Data regarding the right eyes of 23 healthy volunteers were included. The mean blur rate (MBR) of the macula, which represents the choroidal blood flow velocity, was evaluated using LSFG. Intraocular pressure (IOP), systolic blood pressure (SBP), diastolic blood pressure (DBP), mean blood pressure (MBP), ocular perfusion pressure (OPP), and MBR were assessed at baseline, immediately after immersion (0 minutes), and 10, 20, and 30 minutes later.

Results: At 0 minutes, SBP, DBP, MBP, and OPP values were lower than those at baseline. The MBR significantly declined immediately after immersion to -6.0 ± 5.2%. However, there were no changes in these parameters after 10, 20, or 30 minutes. A significant positive correlation was observed between the MBR, SBP, DBP, MBP, and OPP values. In healthy individuals, the dominant parasympathetic activity induced by warm stimulation reduced the choroidal hemodynamic rate in the macula and decreased systemic circulatory dynamics, which normalized after 10 minutes.

Conclusions: These findings suggest that the dominant parasympathetic activity induced by warm water immersion at 40°C may lead to a reduction in the systemic circulation rate and choroidal blood flow rate in the macula. These findings may help prevent and treat various retinal choroidal diseases, in which sympathetic hyperactivity is involved in the pathogenesis of the disease.

## Introduction

The autonomic nervous system includes the sympathetic and parasympathetic systems. Cold stress activates the sympathetic nervous system and the release of norepinephrine, resulting in increased blood pressure (BP), heart rate (HR), and decreased choroidal thickness, measured with enhanced depth imaging optical coherence tomography. These findings are referred to as pressor responses and cold pressor tests are used to evaluate sympathetic responses in systemic cardiovascular dynamics [1-6]. This common stress test helps evaluate cardiac autonomic function and is used as an experimental pain stimulus in the cooling of the hands, feet, or the entire body. The foot cooling test differs from the hand and whole-body cooling tests in that both BP and HR values increase, indicating that sudden exposure of the feet to the cold may be experienced as particularly stressful [3]. Meanwhile, a report of a hot water test, wherein water was heated to 47°C, showed that elevated BP is due to an increase in sympathetic nerve activity caused by the pain [7]. In fact, temperatures of 43°C or higher may cause heat-induced pain [8].

In contrast, thermotherapy provides physical benefits such as increased skin temperature and blood flow and psychological benefits such as pain relief and comfort, which can reduce pain and anxiety levels [9,10]. Warm stimulation at 42°C resulted in some improvements in subjective experiences, such as feeling refreshed, with a lowering of muscle stiffness and fatigue levels compared to the corresponding states without any stimulation [10]. Furthermore, nasal mucosal temperature values observed after foot warming are mediated by a neural reflex due to the loss of sympathetic activation in the nasal vascular system, which might be affected by long-acting parasympathetic mediators [11]. Previous reports suggest that appropriate warming stimulation may change systemic and choroidal circulatory dynamics by increasing parasympathetic dominance.

The choroid is a blood-rich tissue of the intraocular blood flow and is characterized by poor autoregulation [12-15]. Interestingly, the choroidal neurons receive sympathetic and parasympathetic innervations and are assumed to play a role in choroidal blood flow regulation [16]. Laser speckle flowgraphy (LSFG) is a noninvasive quantitative measurement of choroidal blood flow that has been used in various studies for normal eyes and retinochoroidal diseases [17-23]. The mean blur rate (MBR) is a quantitative index of relative blood flow velocity. The MBR is significantly higher during the daytime, winter, and mid-luteal phases of the menstrual cycle when sympathetic activity is dominant, than during the nighttime, summer, and late follicular phases of the menstrual cycle when parasympathetic activity is dominant [20-22].

A recent study has shown that increased sympathetic nervous system activation elicited by the cold pressor test in healthy young individuals increased the macular MBR alongside an increase in systemic circulatory dynamics [23]. However, changes in choroidal blood flow using LSFG monitoring after warm stimulation that increases parasympathetic activity have not been evaluated previously. Herein, we aimed to examine the course of changes in choroidal blood flow velocity in the macula after warm water immersion at 40°C with LSFG.

## Materials and methods

Participants

This study was approved by the Ethics Committee of Fukuoka International University of Health and Welfare (approval ID: 20-fiuhw-022) and adhered to the tenets of the Declaration of Helsinki. Written informed consent was obtained from all participants. In this prospective investigation, the right eyes of 23 healthy individuals (nine men and 14 women) without ophthalmic or cardiovascular disease were included (Table 1).

**Table 1:** Characteristics and changes in ocular biometric parameters at baseline and after warm water immersion in participants M: male; F: female; IOP: intraocular pressure; OPP: ocular perfusion pressure; MBR: mean blur rate; min: minutes; SD: standard deviation

Case	Age (years)	Sex	IOP (mmHg)					OPP (mmHg)					MBR					MBR（%）				
			Baseline	0 min	10 min	20 min	30 min	Baseline	0 min	10 min	20 min	30 min	Baseline	0 min	10 min	20 min	30 min	Baseline	0 min	10 min	20 min	30 min
1	21	F	14.3	14.3	15.0	12.0	12.3	45.3	43.5	41.2	44.2	46.8	11.4	10.9	11.3	11.5	12.0	100.0	95.6	99.1	100.9	105.3
2	20	F	11.3	12.7	11.0	10.7	11.7	48.3	40.4	39.9	47.5	41.9	12.7	11.4	12.6	13.8	12.8	100.0	89.8	99.2	108.7	100.8
3	22	F	13.0	12.3	12.0	13.7	13.3	42.8	40.8	45.6	41.6	43.8	8.4	7.9	8.2	8.7	8.9	100.0	94.0	97.6	103.6	106.0
4	20	F	11.3	11.0	10.0	10.7	12.3	34.0	34.6	36.9	35.1	35.9	26.8	26.8	25.1	25.8	28.1	100.0	100.0	93.7	96.3	104.9
5	22	F	12.3	11.7	11.7	12.0	11.0	42.1	37.6	39.2	38.2	44.3	7.4	7.0	7.0	7.3	8.0	100.0	94.6	94.6	98.6	108.1
6	21	Ｆ	15.0	14.3	12.3	13.3	12.7	36.3	35.5	37.7	40.7	39.1	23.3	23.0	23.3	23.0	24.3	100.0	98.7	100.0	98.7	104.3
7	22	Ｆ	11.7	12.0	12.0	13.0	12.3	41.4	38.9	40.7	40.6	42.6	21.7	20.6	21.8	20.9	21.4	100.0	94.9	100.5	96.3	98.6
8	22	Ｆ	15.0	13.0	12.7	13.0	14.0	31.9	31.0	30.9	32.8	35.6	9.0	8.5	8.8	8.7	9.1	100.0	94.4	97.8	96.7	101.1
9	20	Ｆ	13.3	13.7	14.3	14.3	13.3	40.0	31.9	34.6	35.9	36.5	12.7	11.8	11.5	10.4	11.3	100.0	92.9	90.6	81.9	89.0
10	45	Ｍ	18.3	19.0	19.0	18.0	19.3	48.1	43.9	43.2	48.7	45.1	7.1	6.3	7.1	6.9	6.7	100.0	88.7	100.0	97.2	94.4
11	21	Ｆ	17.3	15.7	15.0	15.3	13.0	41.4	43.2	43.2	40.9	45.9	12.4	11.6	12.2	11.9	11.5	100.0	93.5	98.4	96.0	92.7
12	28	Ｍ	17.7	17.3	17.0	18.3	16.0	46.3	44.3	44.6	42.4	41.6	18.0	17.6	18.5	17.5	17.8	100.0	97.8	102.8	97.2	98.9
13	20	Ｆ	11.0	11.0	11.0	12.7	12.0	51.9	45.0	53.4	49.5	53.8	40.9	38.5	41.9	40.8	46.8	100.0	94.1	102.4	99.8	114.4
14	37	Ｍ	15.3	15.3	15.0	15.3	17.7	44.0	41.8	41.0	44.7	41.2	11.4	11.2	11.4	11.3	10.1	100.0	98.2	100.0	99.1	88.6
15	43	Ｍ	12.7	14.3	12.7	13.3	13.7	54.4	51.0	53.1	56.5	51.0	9.2	8.9	8.9	9.1	8.5	100.0	96.7	96.7	98.9	92.4
16	20	Ｆ	11.7	9.7	10.3	10.0	10.7	38.7	39.0	37.5	42.0	35.5	14.2	14.0	13.9	14.3	13.5	100.0	98.6	97.9	100.7	95.1
17	22	Ｆ	10.3	10.3	10.0	8.3	9.7	46.1	41.5	46.9	46.1	46.5	19.6	16.5	18.3	18.0	19.0	100.0	84.2	93.4	91.8	96.9
18	21	Ｍ	13.3	12.0	10.0	10.7	11.7	46.3	43.3	45.8	47.3	47.2	13.9	13.7	12.8	11.2	14.0	100.0	98.6	92.1	80.6	100.7
19	21	Ｍ	13.0	12.7	11.7	13.3	12.7	43.0	42.6	46.7	45.1	40.9	15.4	15.0	15.4	15.8	13.7	100.0	97.4	100.0	102.6	89.0
20	20	Ｆ	13.0	11.3	10.3	10.3	10.7	48.6	45.8	48.8	50.4	50.9	29.5	22.5	25.7	28.2	29.1	100.0	76.3	87.1	95.6	98.6
21	21	Ｍ	15.3	17.3	17.0	14.7	17.7	43.6	38.3	39.4	41.5	41.9	9.5	9.0	9.0	9.1	9.4	100.0	94.7	94.7	95.8	98.9
22	21	Ｍ	14.3	12.3	13.0	13.7	12.7	38.4	33.5	33.2	37.0	36.4	14.7	13.6	14.7	14.4	14.4	100.0	92.5	100.0	98.0	98.0
23	21	Ｍ	9.7	9.7	8.7	8.7	9.0	60.5	56.3	60.0	55.7	62.8	14.8	14.1	15.0	14.3	14.5	100.0	95.3	101.4	96.6	98.0
Mean	23.9		13.5	13.2	12.7	12.8	13.0	44.1	41.0	42.8	43.7	43.8	15.8	14.8	15.4	15.3	15.9	0.0	94.0	97.4	97.0	98.9
SD	7.3		2.3	2.4	2.6	2.5	2.5	6.4	5.8	6.8	6.0	6.5	8.1	7.4	8.0	8.0	9.1	0.0	5.2	3.9	6.0	6.4

Each individual was examined for best-corrected visual acuity (BCVA), fundus photography, intraocular pressure (IOP), BP, HR, and LSFG.

Warm water immersion

Examinations were performed at room temperature (24 ± 1°C) and a humidity of 47 ± 3% in a quiet examination room [18,19,21]. During warm water immersion, both feet were immersed up to the ankle in water at 40°C for 60 seconds. The IOP, BP, HR, and LSFG were measured at baseline, immediately after immersion (0 minutes), and 10, 20, and 30 minutes later. All tests were conducted in a sitting position, and each measurement session lasted approximately three minutes [21]. In addition, each participant was instructed to avoid smoking or exercising for at least two hours prior to testing and to rest for 10 minutes in the examination room [19,21].

Laser speckle flowgraphy measurement

**Figure 1:**
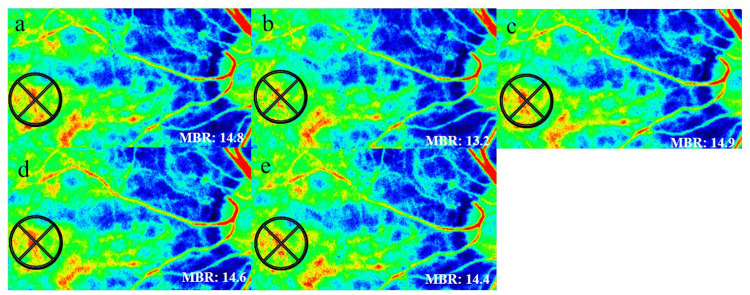
Composite color map image of the mean blur rate (MBR) measured by laser speckle flowgraphy (LSFG) at baseline (a), 0 minutes (immediately after immersion) (b), and at 10 (c), 20 (d), and 30 (e) minutes after the test. The MBR within the region of interest immediately after warm water immersion (b) decreased compared to that at baseline (a). However, after 10 minutes or more (c-e) of immersion, the values returned to baseline.

The LSFG-NAVI (Softcare Ltd., Fukuoka, Japan) was used to measure the hemodynamics of the posterior fundus. The LSFG uses an 830-nm diode laser to illuminate the fundus and detect moving red blood cells in the deep choroidal vessels. Each LSFG measurement was taken three times at baseline, immediately after immersion (0 minutes), and every 10 minutes up to 30 minutes after immersion. Changes in choroidal blood flow velocity were evaluated by excluding large retinal vessels in the macula (Figure 1).

During the follow-up, each circle was automatically set using the LSFG analyzer software (v. 3.0.47; Softcare Ltd., Fukuoka, Japan) at the same site where the circle was set at baseline. The change in the average MBR was evaluated as the change relative to the baseline value, which was set at 100% [15-17, 20-21].

Intraocular pressure and hemodynamics

The IOP, systolic BP (SBP), and diastolic BP (DBP) were examined at baseline, immediately after warm water immersion (0 minutes), and after 10, 20, and 30 minutes. The IOP measurements were performed using a non-contact tonometer. The mean BP (MBP) was calculated from the SBP and DBP, and the ocular perfusion pressure (OPP) was calculated from the MBP and IOP, according to the following equation:

MBP = DBP + 1/3(SBP - DBP) (1)

OPP = 2/3MBP - IOP (2)

Statistics

All data are reported as the mean (standard deviation: SD). Friedman and Scheffe’s paired comparison tests were used for continuous changes in IOP, SBP, DBP, HR, MBP, OPP, and MBR. Spearman’s rank correlation test was utilized to determine the relationship between the rate of change in MBR and other studied factors. P-values of < 0.05 were considered indicative of statistical significance in all tests.

## Results

The average participant age was 23.9 ± 7.3 (range, 20 to 45) years. All participants had a BCVA score of ≥ 1.0 (decimal acuity).

The IOP, SBP, DBP, HR, MBP, and OPP values

Changes in the IOP, SBP, DBP, HR, MBP, and OPP values are summarized in Tables 1-3.

**Table 2:** Changes in systemic factors at baseline and after warm water immersion in participants SBP: systolic blood pressure; DBP: diastolic blood pressure; MBP: mean blood pressure; HR: heart rate; bpm: beats per minute; min: minutes; SD: standard deviation

Case	SBP (mmHg)					DBP (mmHg)					MBP (mmHg)					HR (bpm)				
	Baseline	0 min	10 min	20 min	30 min	Baseline	0 min	10 min	20 min	30 min	Baseline	0 min	10 min	20 min	30 min	Baseline	0 min	10 min	20 min	30 min
1	114	108	111	105	114	77	76	71	74	76	89.3	86.7	84.3	84.3	88.7	86	83	82	81	78
2	114	105	101	116	115	77	67	64	73	63	89.3	79.7	76.3	87.3	80.3	70	63	68	65	68
3	107	107	107	107	109	72	66	76	71	74	83.7	79.7	86.3	83.0	85.7	73	74	72	65	75
4	96	93	93	94	97	54	56	59	56	60	68.0	68.3	70.3	68.7	72.3	74	73	74	74	76
5	103	94	99	98	103	71	64	65	64	73	81.7	74.0	76.3	75.3	83.0	80	80	81	83	81
6	101	96	101	109	105	65	64	62	67	64	77.0	74.7	75.0	81.0	77.7	79	81	83	90	91
7	103	97	103	101	107	68	66	67	70	70	79.7	76.3	79.0	80.3	82.3	82	79	86	83	85
8	93	90	86	88	91	59	54	55	59	66	70.3	66.0	65.3	68.7	74.3	67	66	62	67	71
9	100	85	86	96	98	70	60	67	65	63	80.0	68.3	73.3	75.3	74.7	92	89	94	95	92
10	137	125	128	136	128	81	79	76	82	81	99.7	94.3	93.3	100.0	96.7	76	75	72	69	74
11	116	115	114	105	109	74	75	74	74	78	88.0	88.3	87.3	84.3	88.3	104	106	102	107	104
12	116	113	125	109	115	86	82	76	82	72	96.0	92.3	92.3	91.0	86.3	87	96	89	90	97
13	125	114	126	126	128	79	69	82	77	84	94.3	84.0	96.7	93.3	98.7	70	68	67	68	70
14	113	111	114	114	115	77	73	69	78	75	89.0	85.7	84.0	90.0	88.3	85	81	80	77	74
15	134	128	134	130	129	84	83	81	92	81	100.7	98.0	98.7	104.7	97.0	105	92	87	84	89
16	97	93	91	110	90	65	63	62	62	59	75.7	73.0	71.7	78.0	69.3	85	81	86	92	86
17	110	99	106	101	105	72	67	75	72	74	84.7	77.7	85.3	81.7	84.3	100	93	102	92	97
18	118	113	111	109	119	75	68	70	76	73	89.3	83.0	83.7	87.0	88.3	84	78	81	80	77
19	112	109	117	119	113	70	70	73	72	64	84.0	83.0	87.7	87.7	80.3	81	78	82	79	78
20	125	115	124	121	123	76	71	71	76	77	92.3	85.7	88.7	91.0	92.3	74	68	77	75	79
21	117	110	110	109	118	74	70	72	72	75	88.3	83.3	84.7	84.3	89.3	89	85	85	79	88
22	109	100	100	110	103	64	53	54	59	59	79.0	68.7	69.3	76.0	73.7	74	69	73	75	65
23	126	119	127	120	125	95	89	91	85	99	105.3	99.0	103.0	96.7	107.7	89	81	79	83	84
Mean	112.4	106.0	109.3	110.1	111.3	73.3	68.9	70.1	72.1	72.2	86.3	81.3	83.2	84.8	85.2	82.9	80.0	81.0	80.6	81.7
SD	11.7	11.3	13.8	11.6	11.3	8.9	9.0	8.7	8.8	9.4	9.4	9.4	9.9	9.1	9.4	10.4	10.3	10.1	10.5	10.1

**Table 3:** Changes in ocular biometric parameters and systemic factors at baseline and after the warm water immersion Values are presented as mean±SD SD: standard deviation; min: minutes; IOP: intraocular pressure; SBP: systolic blood pressure; DBP: diastolic blood pressure; HR: heart rate; MBP: mean blood pressure; bpm: beats per minute; OPP: ocular perfusion pressure; MBR: mean blur rate *** P < 0.001; ** P < 0.01; * P < 0.05.

		After the warm water immersion				Friedman test (P-value)	Scheffe’s paired comparison (P-value)			
	Baseline	0 min	10 min	20 min	30 min		0 min	10 min	20 min	30 min
IOP (mmHg)	13.5±2.3	13.2±2.4	12.7±2.6*	12.8±2.5	13.0±2.5	0.021	0.935	0.032	0.593	0.998
SBP (mmHg)	112.4±11.7	106.0±11.3***	109.3±13.1	110.1±11.6	113.3±11.3	<0.001	<0.001	0.353	0.242	1.000
DBP (mmHg)	73.3±8.9	68.9±9.0**	70.1±8.7	72.1±8.8	72.2±9.4	0.001	0.007	0.172	0.909	0.983
HR (bpm)	82.9±10.4	80.0±10.3	81.0±10.1	80.6±10.5	81.7±10.1	0.108	0.177	0.801	0.801	0.997
MBP (mmHg)	86.3±9.4	81.3±9.4***	83.2±9.9	84.8±9.1	85.2±9.4	<0.001	<0.001	0.094	0.663	0.974
OPP (mmHg)	44.1±6.4	41.0±5.8***	42.8±6.8	43.7±6.0	43.8±6.5	<0.001	0.008	0.667	0.997	0.999
MBR	15.8±8.1	14.8±7.4	15.4±8.0	15.3±8.0	15.9±9.1	<0.001	<0.001	0.318	0.090	0.604
MBR (%)	100.0±0.0	94.0±5.2	97.4±3.9	97.0±6.0	98.9±6.4	<0.001	<0.001	0.318	0.090	0.604

The SBP, DBP, MBP, and OPP values significantly decreased immediately after warm water immersion compared to those at baseline. They did not change thereafter (Table 3). The IOP decreased significantly only 10 minutes after the test, and the HR did not change throughout the study (Table 3).

The LSFG data

The changes in MBR are shown in Tables 1 and 3. The mean MBR values at baseline, immediately after warm water immersion (0 minutes), and 10 minutes or later thereafter were 15.8 ± 8.1, 14.8 ± 7.4, 15.4 ± 8.0, 15.3 ± 8.0, and 15.9 ± 9.1, respectively. The MBR values significantly decreased by -6.0 ± 5.2% at 0 minutes, but at 10, 20, and 30 minutes, they were comparable to baseline values (Table 3).

Correlation between MBR and IOP, SBP, DBP, HR, and OPP

The rate of change in MBR was correlated with the change in IOP, SBP, DBP, HR, and OPP from baseline to immediately after warm water immersion. The MBR was significantly correlated with SBP, DBP, MBP, and OPP values. However, MBR did not correlate significantly with IOP and HR (Table 4).

**Table 4:** Relationship between MBR and other studied factors MBR: mean blur rate; IOP: intraocular pressure; SBP: systolic blood pressure; DBP: diastolic blood pressure; HR: heart rate; MBP: mean blood pressure; OPP: ocular perfusion pressure

	MBR	
	Coefficient	P-value
IOP	-0.111	0.612
SBP	0.594	0.002
DBP	0.488	0.018
HR	0.161	0.461
MBP	0.640	<0.001
OPP	0.560	0.005

## Discussion

In the present study, the SBP, DBP, MBP, OPP, and MBR values reduced significantly after foot immersion in warm water at 40°C for 60 seconds, with no further changes observed after 10 minutes or later. Furthermore, the MBR had statistically significant positive correlations with SBP, DBP, MBP, and OPP.

Central serous chorioretinopathy (CSC) is an ocular disease precipitated by increased sympathetic nervous system activity and a stressful personality and has been reported to completely resolve subretinal fluid within six months. The BCVA significantly improved at six months compared to the initial BCVA, and the average macular MBR significantly decreased at three and six months, respectively, compared to baseline. Thus, macular choroidal blood flow velocity decreases concurrently with CSC regression [17]. In addition, MBR is significantly reduced with decreased systemic circulatory activity during the nighttime, summer, and late follicular phases of the menstrual cycle, when parasympathetic activity is dominant, compared to the daytime, winter, and mid-luteal phases of the menstrual cycle [20-23]. In this study, choroidal blood flow velocity was significantly reduced immediately after ankle immersion in 40°C warm water due to increased parasympathetic activity. Thus, the results of the present study, along with those of previous reports, indicate that choroidal blood flow velocity is reduced when the parasympathetic nervous system is dominant.

The temperature at which heat-induced pain occurs, where the sympathetic nervous system dominates, is considered to be 43°C or higher [8]. In water heated to 47°C, pain increases sympathetic nervous system activity and BP [7]. In contrast, the warm stimulation condition at 42°C correlated with improvements to subjective complaints such as refreshed feelings and relief of muscle stiffness and fatigue compared to those reported by the control group [10]. Furthermore, foot baths in water at a temperature ranging from 41 to 43°C may result in transient improvements in systemic arterial stiffness in healthy women [24]. The elevation of nasal mucosal temperature following foot warming is mediated by a neural reflex due to the loss of sympathetic nervous system activation in the nasal vascular system and a potential additive contribution of the long-acting parasympathetic mediators [11].

Overall, the evidence from the previous and present reports suggests that warm stimulation at approximately 40°C, where parasympathetic activity is dominant, reduces choroidal blood flow velocity, following a decrease in systemic hemodynamics. In addition, these results may help prevent and treat various retinal choroidal diseases, such as CSC, in which sympathetic hyperactivity is involved in the pathogenesis of the disease.

There are some limitations to this study. First, the participants were mainly young, healthy adults, and the only stimulus condition was immersion of both feet up to the ankle in water at 40°C for 60 seconds. Second, because of measurement time constraints, choroidal thickness examination and evaluation have not been performed. Further research should examine how elevated parasympathetic activity affects choroidal changes in healthy individuals of different sexes and ages, considering various stimulus conditions.

## Conclusions

Our data suggest that in healthy individuals, the dominant parasympathetic activity induced by warm water immersion reduces macular choroidal blood flow velocity as well as systemic dynamics. These new observations suggest that the LSFG may provide a new method for evaluating parasympathetic nerve activation and intrinsic vascular reactivity in the eye. Future research should examine whether parasympathetic dominance contributes to preventing CSC onset or improving symptoms.
